# Analysis and Functional Consequences of Increased Fab-Sialylation of Intravenous Immunoglobulin (IVIG) after Lectin Fractionation

**DOI:** 10.1371/journal.pone.0037243

**Published:** 2012-06-04

**Authors:** Fabian Käsermann, David J. Boerema, Monika Rüegsegger, Andreas Hofmann, Sandra Wymann, Adrian W. Zuercher, Sylvia Miescher

**Affiliations:** 1 Research & Development, CSL Behring AG, Bern, Switzerland; 2 Research & Development, CSL Behring LLC, Kankakee, Illinois, United States of America; Oxford University, United Kingdom

## Abstract

It has been proposed that the anti-inflammatory effects of intravenous immunoglobulin (IVIG) might be due to the small fraction of Fc-sialylated IgG. In this study we biochemically and functionally characterized sialic acid-enriched IgG obtained by *Sambucus nigra* agglutinin (SNA) lectin fractionation. Two main IgG fractions isolated by elution with lactose (E1) or acidified lactose (E2) were analyzed for total IgG, F(ab’)_2_ and Fc-specific sialic acid content, their pattern of specific antibodies and anti-inflammatory potential in a human *in vitro* inflammation system based on LPS- or PHA-stimulated whole blood. HPLC and LC-MS testing revealed an increase of sialylated IgG in E1 and more substantially in the E2 fraction. Significantly, the increased amount of sialic acid residues was primarily found in the Fab region whereas only a minor increase was observed in the Fc region. This indicates preferential binding of the Fab sialic acid to SNA. ELISA analyses of a representative range of pathogen and auto-antigens indicated a skewed antibody pattern of the sialylated IVIG fractions. Finally, the E2 fraction exerted a more profound anti-inflammatory effect compared to E1 or IVIG, evidenced by reduced CD54 expression on monocytes and reduced secretion of MCP-1 (CCL2); again these effects were Fab- but not Fc-dependent. Our results show that SNA fractionation of IVIG yields a minor fraction (approx. 10%) of highly sialylated IgG, wherein the sialic acid is mainly found in the Fab region. The tested anti-inflammatory activity was associated with Fab not Fc sialylation.

## Introduction

Replacement therapy with plasma-derived immunoglobulin G (IgG) is the standard of care to treat primary and secondary immunodeficiency. For this purpose IgG is applied either intravenously (IVIG) or subcutaneously (SCIG). IVIG/SCIG is prepared from large plasma pools from more than 10′000 donors, which ensures a diverse antibody repertoire. Additionally, over the years IVIG/SCIG has been increasingly used for immunomodulation of acute and chronic autoimmune diseases (for an overview see ref [1]). Commonly treated disorders include idiopathic thrombocytopenic purpura (ITP), Kawasaki disease, Guillain-Barré syndrome, chronic inflammatory demyelinating polyneuropathy (CIDP), myasthenia gravis and several rare diseases; several other indications are currently under investigation [2]–[6]. Despite the wide use of IVIG, its mechanism of action is still not fully understood. A number of possible non-exclusive mechanisms have been proposed to explain the immunomodulatory effects of IVIG. They include interference with complement components and the cytokine network, modulation of B and T cell function, Fc receptor blockage and effects on the anti-idiotype network. Probably there are multiple pathways operating in parallel [7]–[11].

In autoimmune and inflammatory diseases, patients are treated with high doses of IVIG in the range of 1–2 g/kg bodyweight. The need for these high doses might be explained by a limited amount of the active component present in IVIG. Identification and enrichment of such a putative “active fraction” would potentially allow development of a product with improved efficacy. In a series of studies from the group of Jeffrey Ravetch, the small fraction of Fc-sialylated IgG was proposed as a constituent of IVIG with increased protective effect in a mouse model of rheumatoid arthritis (K/BxN) [12]–[15]. They showed that a subfraction of IVIG enriched for sialic acid by lectin affinity fractionation with the sialic acid specific lectin *Sambucus nigra* agglutinin (SNA), had ten times higher efficacy in the K/BxN model [12]. Subsequently, using recombinant Fc fragments that were highly sialylated by *in vitro* enzymatic glycoengineering (S+ Fc), the component responsible for the anti-inflammatory effects in the K/BxN model was identified as α2,6-linked terminal sialic acid in the Fc region of IgG [14] and reviewed in [16]. Based on a series of sophisticated experiments, a new mechanism triggered by the sialylated Fc region in IVIG binding to DC-SIGN on myeloid regulatory cells resulting in secretion of IL-33 was proposed. The increased IL-33 level apparently stimulates the expansion of IL-4 producing basophils leading to an increased expression of the inhibitory Fc receptor FcγIIB on effector macrophages and to the suppression of the K/BxN serum induced arthritis [15]. In this study we aimed to test if the effects observed so far only in the K/BxN mouse model, could be reproduced in an *in vitro* human system and if the proposed Fc-sialylation dependent mechanism contributes in general to the overall anti-inflammatory effect of IVIG.

## Results

### Production of Sialic Acid-enriched IVIG by Lectin Affinity Chromatography

**Figure 1:**
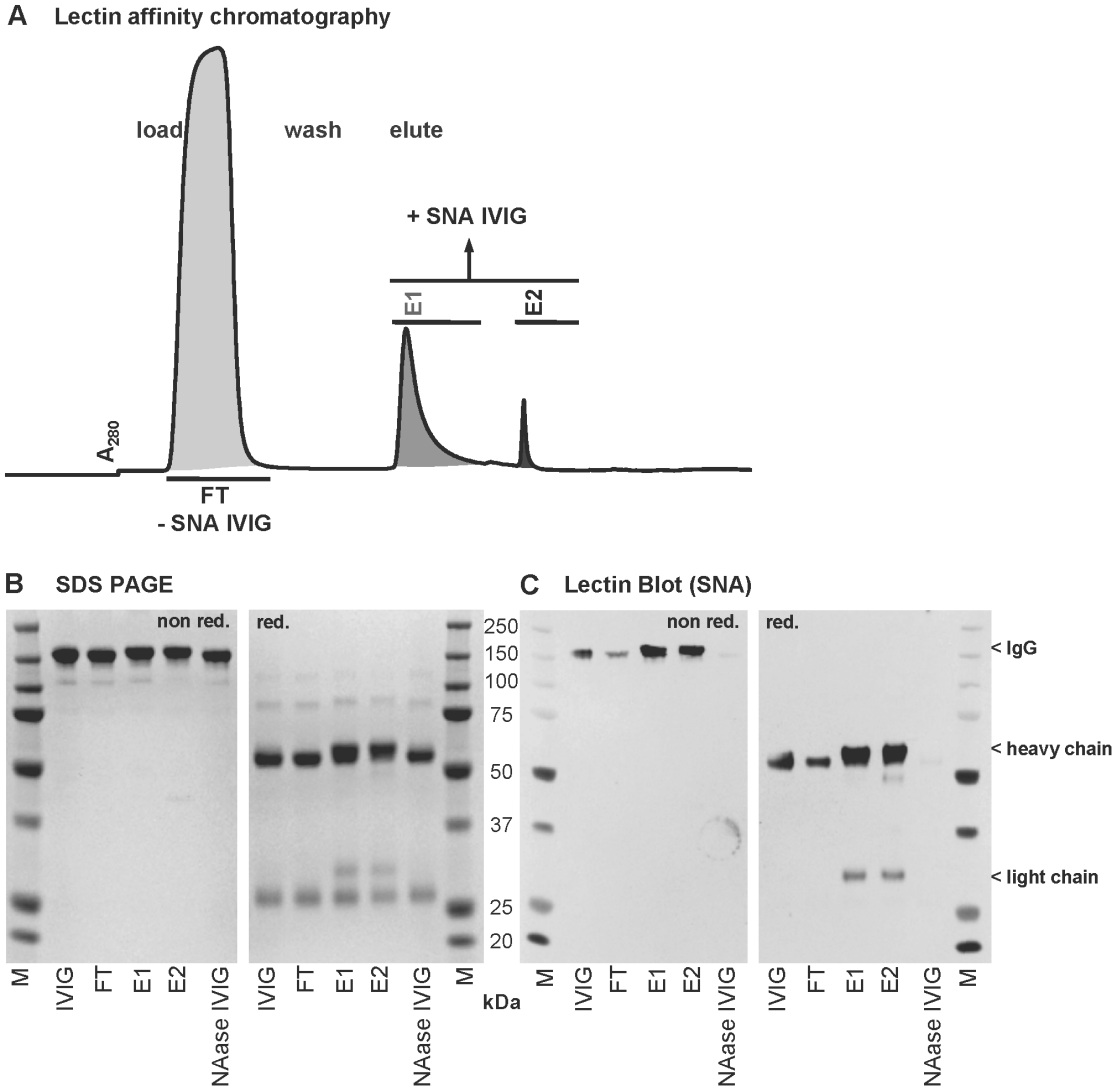
Lectin affinity chromatography. A: Typical fractionation chromatogram of 1 gram IVIG on a 90 mL SNA-agarose column, showing the SNA non-binding (-SNA IVIG) flowthrough fraction (FT) and the SNA binding fractions (+SNA IVIG) eluate 1 (E1) and eluate 2 (E2). B: SDS PAGE and C: Lectin Blot (SNA) analysis of the SNA-purified IVIG fractions. IVIG: source IVIG before fractionation, FT: SNA non-binding flowthrough, E1: SNA binding eluate 1, E2: SNA binding eluate 2, NAase IVIG: Neuraminidase treated (desialylated) IVIG, M: molecular marker, red.: gel run under reducing conditions, non red.: gel run under non-reducing conditions.

In earlier studies lectin chromatography with sialic acid-specific *Sambucus nigra* agglutinin (SNA) was applied to produce highly sialylated IVIG fractions [12], [17], [18]. We adapted this method by up-scaling and sub-fractionating the elution fractions. Instead of combining the eluted SNA+ fractions in one pool, the fractions obtained by elution with neutral lactose (elution fraction 1; E1) and by elution with acidic lactose (elution fraction 2; E2) were collected and analyzed separately (Fig. 1A). This process yielded on average 8–10% of the loaded IVIG in E1 and 1.5–2% in E2.

### Characterization by SDS-Page and Lectin Blotting

Sialylation of the different fractions was assessed by SDS-PAGE under non-reducing and reducing conditions (Fig. 1B), followed by lectin blotting with biotinylated SNA (Fig. 1C). A strong enrichment of sialylated IgG was observed in E1 and E2 when the SDS PAGE was run under non-reducing conditions (Fig. 1C, left panel). These results are in agreement with results reported by Kaneko et al. [12]. However, when the electrophoresis was performed under reducing conditions, we found that in addition to the Ig heavy chains, there was a clear enhancement of sialic acid content in the Ig light chains of E1 and E2. This indicated that IgG sialylated in the Fab-region was enriched by lectin chromatography (Fig. 1C, right panel). In addition, the flow-through fraction (FT) showed a weak positive signal in the lectin blot indicating that the FT was not free of sialylated IgG and therefore could not be used as a “sialic acid free” control preparation. If the FT was re-applied to an SNA column no additional material was recovered in the binding fractions, indicating that the affinity column had not been overloaded, but rather that the remaining sialic acid might not be accessible for SNA (data not shown).

**Figure 2:**
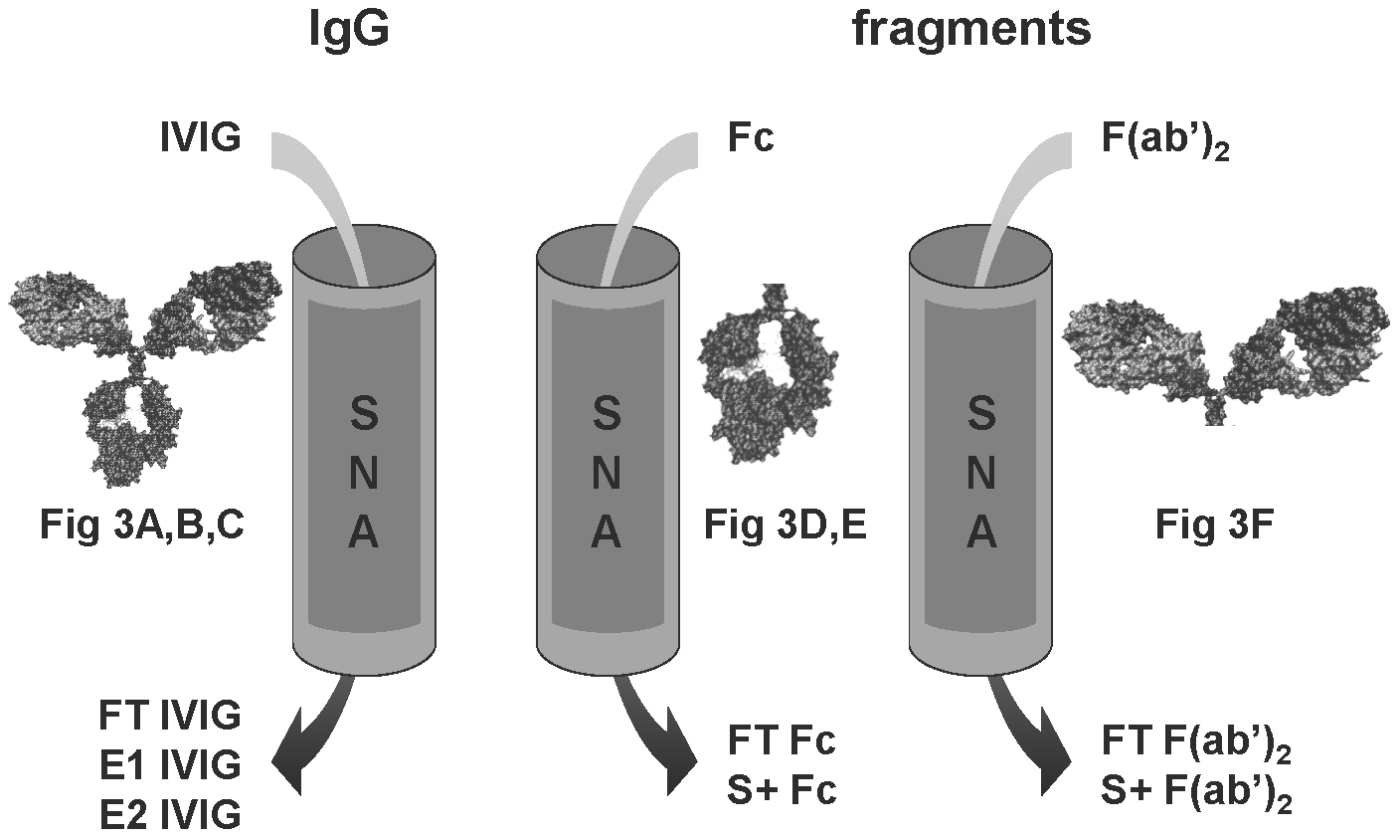
Overview of SNA affinity chromatography with IgG, Fc and F(ab’)_2_ fragments. Schematic presentation of purification strategy showing various starting materials for SNA-chromatography and resulting fractions.

Based on these findings, IgG and various fractions thereof were enzymatically completely desialylated using neuraminidase to produce non-sialylated control samples (NAase IVIG). The same lectin affinity purification method was then applied to different starting materials finally providing enriched sialylated Fc fragments (S+ Fc) from plasma Fc and sialylated F(ab’)_2_ (S+ F(ab’)_2_) from plasma F(ab’)_2_ (Fig. 2).

### Characterisation by HPLC and LC-MS

**Figure 3:**
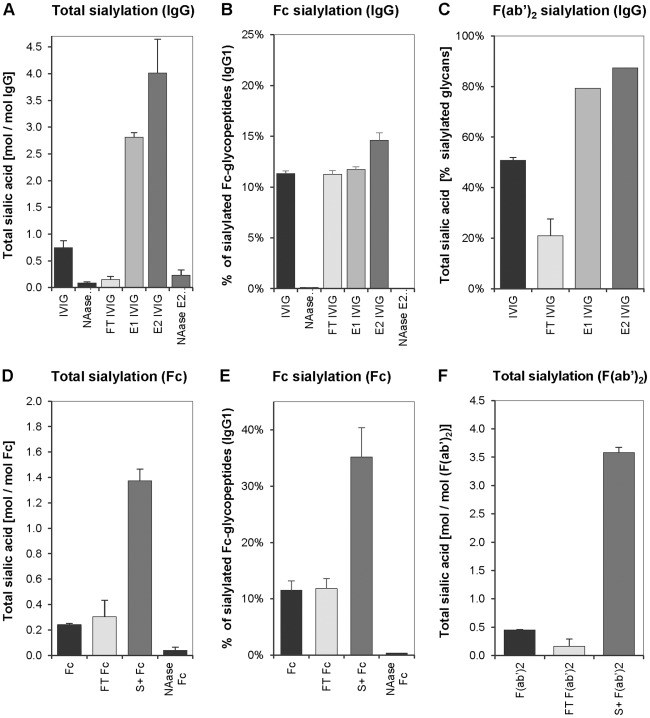
Analysis of total and Fc sialylation by HPLC and LC-MS. Upper row: sialylation in lectin purified IVIG. Lower row: sialylation in IgG fragments. A: Total sialic acid in IgG of the indicated fractions by HPLC. B: Percentage of sialylated IgG1 Fc-glycopeptides in the indicated IgG fractions (LC-MS). C: Percentage of sialylated glycans in F(ab’)_2_ fragments produced from the indicated IgG fractions (LC-MS). D: Total sialic acid content in fractions from Fc run over the lectin column (HPLC). E: Percentage of sialylated IgG1 Fc-glycopeptides in the indicated Fc fractions (LC-MS). F: Total sialic acid content in F(ab’)_2_ fragments purified over the lectin column (HPLC). Mean values and standard deviations measured from three independently produced batches are shown. NAase: Neuraminidase treated, S+ Fc: SNA binding fraction of Fc fragments, S+ F(ab’)_2_: SNA binding fraction of F(ab’)_2_ fragments. FT: flow through (SNA non-binding), E1/E2 IVIG: SNA binding fractions of IVIG.

Sialylation levels of starting materials, fractions obtained by SNA-chromatography and by desialylation with neuraminidase were characterized by HPLC and LC-MS techniques. In Figure 3 the top row shows sialylation patterns measured from IVIG that was purified by lectin affinity chromatography (Fig. 3A, B, C). In the lower row sialic acid contents of fragments purified on the lectin column are shown (Fig. 3D, E, F). In one approach total sialic acid (Neu5Ac) was quantified by HPLC; results were expressed as mol sialic acid per mol IgG. Total sialic acid was enriched in E1 and even more substantially in E2 IVIG, compared to the respective starting material and the non-binding FT fractions (Fig. 3A). A similar enrichment of total sialic acid was observed when purified Fc and F(ab’)_2_ fragments from plasma IgG were enriched via SNA chromatography to yield S+ Fc (Fig. 3D) and S+F(ab’)_2_ (Fig. 3F), respectively. In all preparations, treatment with neuraminidase effectively removed sialic acid residues (Fig. 3A, B, D, E).

Fc-specific sialylation was assessed by LC-MS. We found a substantial enrichment of sialic acid in the eluted fraction when Fc fragments were used as the starting material (Fig. 3E). In contrast, when applying IVIG to the SNA column only a modest increase in Fc-sialylation from 12% to 14% was observed in the SNA-enriched E2 fraction (Fig. 3B) and no enrichment in E1 (Fig. 3B). These data strongly suggested that SNA affinity chromatography preferentially selected for sialic acid residues in the Fab region of IgG whereas only a modest increase in Fc sialylation was achieved with this method. This hypothesis was further supported by a clear enrichment of sialylation in the F(ab’)_2_ region when IVIG was used as starting material for SNA chromatography (Fig. 3C). Measurement of total sialic acid by HPLC in Fc and F(ab’)_2_ fragments from IVIG fractions further confirmed these results (Fig. S1 A&B) and cleavage into Fc and F(ab’)_2_ fragments was confirmed by SDS-PAGE (Fig. S1 C&D). No differences in IgG sialylation were found in IVIG from different manufacturers (data not shown).

### Composition of Specific Antibodies in IVIG, FT, E1 and E2

**Figure 4:**
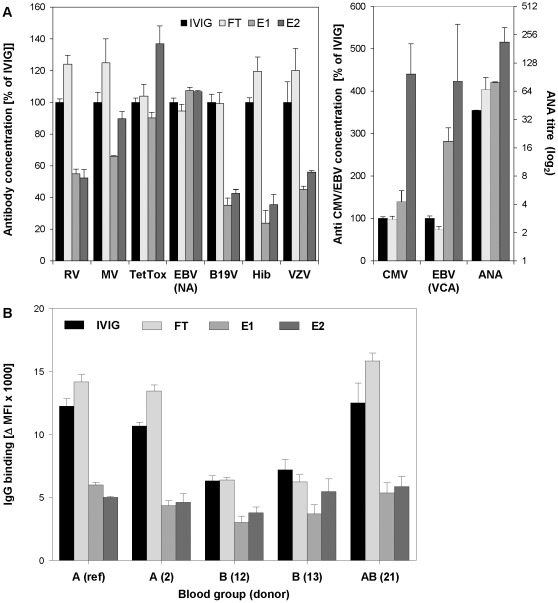
Specific antibody concentrations and human erythrocyte binding. A: Concentrations of antibodies against a panel of antigens. Results are expressed as % of specific concentrations measured in IVIG. RV: rubella virus, MV: measles virus, TetTox: tetanus toxoid, EBV (EBNA): Epstein-Bar virus (nuclear antigen), B19V: human parvovirus B19, Hib: Haemophilius influencae, VZV: varizella zoster virus, CMV: cytomegalovirus, EBV (VCA): Epstein-Barr virus (capsid antigen). Concentrations are normalized to IVIG and shown in linear scale (left axes). ANA: anti-nuclear antibodies, absolute titres are shown in a log_2_ scale (right axis). Mean values and standard deviations measured from three independently produced batches are shown. B: Binding of different IVIG fractions to erythrocytes (RBC) from donors with blood group A, B or AB have been analysed by flow cytometry. MFI: mean fluorescence intensity.

To address whether fractionation of IVIG by SNA chromatography might skew the specific antibody pattern, IVIG, FT and sialic acid-enriched fractions E1 and E2 were tested for binding to various antigens. As shown in Figure 4, SNA lectin chromatography modified the distribution of specific antibodies in the E1 and E2 fractions. Whereas some specificities were not or only marginally affected (Fig. 4A; TetTox, EBV (NA)) others were either decreased (Fig. 4A; RV, MV, B19V, Hib, VZV) or increased (Fig. 4A; CMV, EBV (VCA)). Similarly, testing on Hep-2 cells indicated an enrichment of specific anti-nuclear antibodies (Fig. 4A; ANA) in the E2 fraction. No differences in titres were observed with IVIG pre-incubated under the same conditions as E2 (lactose/acetic acid pH 3.5). Furthermore, the anti-CMV titre was not decreased in desialylated E2 (NAase E2), indicating that the antibody-antigen interaction was not sialic acid dependent (data not shown). Binding of IgG to human blood group A and AB erythrocytes was markedly lowered in E1 and E2 compared to unfractionated IVIG and the FT fraction (Fig. 4B), while a less pronounced effect on binding to B erythrocytes was noted. Similar results were obtained with sheep erythrocytes and less pronounced with rabbit erythrocytes (data not shown). Treatment with neuraminidase also did not influence binding of IVIG to human erythrocytes (data not shown). Overall, these findings demonstrate that the composition of specific antibodies was skewed in sialic-acid enriched IgG fractions, supporting the claim that lectin affinity chromatography fractionation of IVIG is mainly driven by Fab-interactions. Subclass distribution in the different fractions was only marginally shifted, with slightly decreased IgG2 in E2 and a modest increase of IgG4 in E1 and E2 (Fig. S2).

### Anti-inflammatory Effects *in vitro*

**Figure 5:**
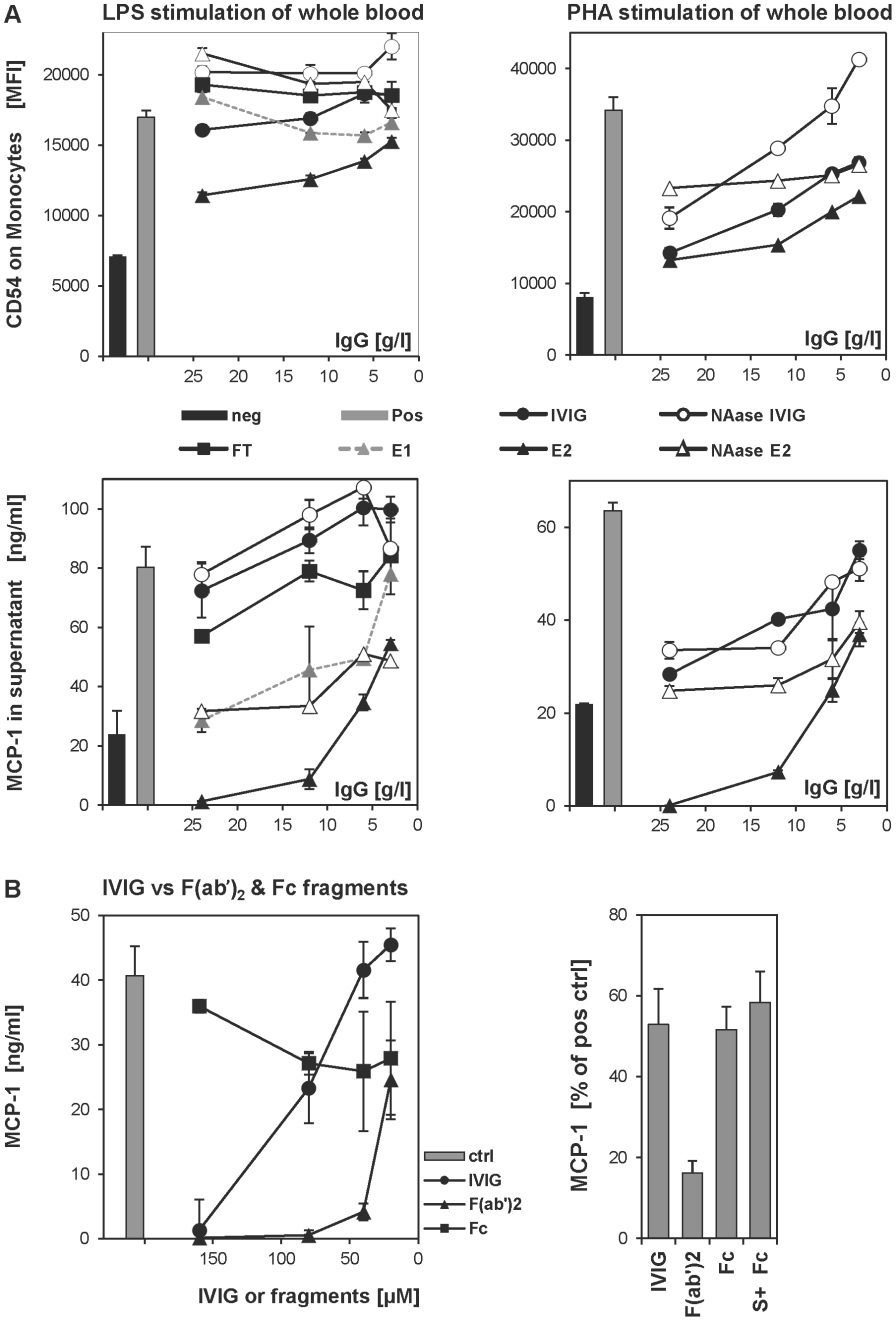
Whole blood stimulation assay. A: Whole blood was stimulated with LPS (left panels) or PHA (right panel). *Upper panels:* concentration dependent inhibition of CD54 (ICAM-1) expression on monocytes by the indicated IVIG fractions was monitored by flow cytometry. MFI: mean fluorescence intensity. *Lower panels:* concentration dependent inhibition of MCP-1 (CCL2) release by IVIG was quantified by ELISA in the supernatant of stimulated blood cultures. Mean values from representative experiments using blood from single donors are shown. neg: no stimulation, pos: stimulation without IVIG, FT: flow through, E1/E2 SNA binding IVIG fractions, NAase: neuraminidase treated. B: Inhibition of PHA mediated MCP-1 release by Fc and F(ab')_2_ fragments. *Left panel:* Mean MCP-1 concentration of stimulated minus non stimulated samples from a representative experiment is shown. *Right panel:* PHA stimulated whole blood was treated with 40 µM IgG, Fc, F(ab’)_2_ or highly sialylated Fc (S+Fc). Normalized values from experiments with blood from two donors are shown. S+ Fc: SNA binding Fc fraction (highly sialylated Fc).

Potential anti-inflammatory effects of IVIG and sialic acid-enriched fractions were assessed in a functional *in vitro* assay (Fig. 5). Whole blood was stimulated with LPS (Fig. 5A left panels) or PHA (Fig. 5A right panels) and cell surface expression of CD54 (ICAM-1) on monocytes as well as MCP-1 secretion into the cell culture supernatant were quantified. Under the chosen conditions the results were most pronounced for MCP-1, but other cytokines were measured as well (IL-6, IL-8, MIP-1β and IL-1ra), yielding similar results (data not shown). LPS induced upregulation of CD54 expression on monocytes; this effect was down-regulated by E2 in a dose-dependent manner (Fig. 5A top left) and could be abrogated by treatment with neuraminidase (NAase E2). Similarly, PHA-induced CD54 expression was inhibited by E2 slightly more efficiently than with all other tested fractions. Again neuraminidase treatment abolished the anti-inflammatory effect (Fig. 5A top right). MCP-1 release into the supernatant was inhibited by E1 and E2 in a dose dependent fashion; the effect was more pronounced than with IVIG (Fig. 5A, bottom panels). Similar as for CD54 expression, the inhibitory effect was lost when E2 was desialylated. Control experiments showed that IVIG and sialic acid-enriched fractions contained anti-LPS antibodies, however, the measured amounts did not correlate with the observed anti-inflammatory effects, suggesting additional mechanisms at work (E2 showed a lower anti-LPS compared to IVIG, data not shown). No antibodies against the PHA used in these experiments were detected. Whereas, in agreement with recently reported data using PHA-L [19], antibodies to PHA from different providers were found in IVIG. Interestingly, inhibition of PHA-induced MCP-1 secretion by IVIG was dependent on the F(ab’)_2_ whereas the Fc region did not appear to contribute to anti-inflammatory effects in this system (Fig. 5B left panel). Similarly, highly sialylated Fc fragments (S+ Fc) tested at 40 µM did not show an inhibitory effect on the PHA-mediated MCP-1 release. This result supports the finding, that IVIG effects are sialic acid, in particular Fc-sialylation independent in this system (Fig. 5B right panel).

## Discussion

In this study we have purified IVIG by lectin chromatography and characterized the resulting highly sialylated IVIG fractions termed E1 and E2. We show that SNA affinity chromatography preferentially enriches for sialylated F(ab’)_2_ fragments, with only a minor accumulation of Fc sialylated IgG molecules. The purification process led to skewing of the antibody pattern. Our functional data from a human *in vitro* inflammation model indicate increased anti-inflammatory activity associated with the Fab sialylated fragments.

In most previous studies using sialylated IgG molecules generated by SNA chromatography no distinction of the E1 and E2 fractions was made. Indeed, in initial studies on the role of IgG sialylation [12]–[14] the E1 and E2 fractions were pooled to yield one single SNA+ fraction. In the recent paper by Guhr et al. [18] the experiments were performed using the E1 fractions. The authors mention that the effect of the E2 fraction was addressed as well yielding identical results [18]. Our present work demonstrates that a separate analysis of E1 and E2 is important, as our data suggest that the highly sialylated E2 fraction contains the highest anti-inflammatory potential, whereas in our hands E1 in most respects was very similar to the unfractionated IVIG.

Analysis of the eluted E1 and E2 fractions by SDS-PAGE under reducing conditions (Fig. 1C) yielded the first indication that SNA chromatography effectively selects for F(ab’)_2_-sialylated IgG molecules. This finding is in agreement with the report by Stadlmann et al. [17]. We confirm and extend this initial finding by HPLC and LC-MS analyses (Fig. 3 and S1). Our data comparing IgG, Fc and F(ab’)_2_ fragments as starting materials for SNA chromatography even indicate a preferential selection of F(ab’)_2_-sialylated molecules. However, when using Fc fragments as the starting material sialylated fragments are effectively enriched (Fig. 3D, E), but when the complete IgG molecule is applied to an SNA column the enrichment of Fc-sialylated IgG is minor (Fig. 3B). We speculate that the sialic acid residues of the Fab region are more readily accessible for binding to column-bound SNA lectins and therefore effectively compete out the sialic acid in the Fc region. Our data support earlier findings [17].

ELISA analysis shows that SNA chromatography skews the antibody pattern of the eluted fraction compared to the starting material (Fig. 4). Our data indicate that this skewing is probably not due to preferential accumulation or depletion of a specific IgG subclass, as only a slight depletion of IgG2 and a small accumulation of IgG4 was noted (Fig. S2). It can be speculated that the glycosylation profile of F(ab’)_2_ including the sialic acid residues nevertheless contributes to and thus can influence the binding site of a particular antibody. This may also play a role in idiotype-anti-idiotype interactions. Even though we cannot explain why for some antigens binding is increased and decreased for others, our data are in line with the finding that IgG selection by SNA chromatography is largely F(ab’)_2_ dependent.

We tested the anti-inflammatory effects of unfractionated IVIG *vs.* SNA-binding IVIG fractions in a human whole blood assay, using CD54 and MCP-1 (CCL2) as two markers for inflammatory responses. Other cytokines (e.g. IL-6, IL-8, MIP-1β, IL-1ra) yielded similar results (data not shown), however under the chosen conditions the effects were most clearly observed with MCP-1. We induced inflammation *in vitro* using two different, well established polyclonal inflammatory stimuli LPS and PHA and focused on early/rapid responses after over-night stimulation. We found that anti-inflammatory effects were most robust with E2 (Fig. 5A). Again this effect was F(ab’)_2_ but not Fc dependent (Fig. 5B). To our knowledge no similar data using an *in vitro* model of human inflammation have been published to date. In earlier studies effective binding of SNA+ IgG to human B-cells via CD22 [20] and S+ Fc binding to human DC-SIGN expressed on CHO cells [13] was shown; however, no further functional consequences in a human system have been presented in these papers. In our *in vitro* studies great care was taken to assure that all preparations were endotoxin/LPS free. It is known that prevention of LPS contamination of such material prepared at lab scale is challenging and therefore it is essential to exclude that contaminating LPS influences the results and leads to erroneous interpretations. We also addressed whether anti-LPS or anti-PHA antibodies present in IVIG might account for the anti-inflammatory effect. Indeed, anti-LPS antibodies were found in IVIG, however, the content did not correlate with the observed anti-inflammatory effect, thus excluding that this might explain the observed inhibitory effects.

The data presented in this manuscript demonstrate an improved anti-inflammatory effect of highly sialylated IgG (E2 fraction) in the tested system; however, our results do not indicate that this is mediated by Fc-sialylation but rather is dependent on F(ab’)_2_ sialylation. This seems to be in contrast with data from the Ravetch group [12]–[15] claiming that Fc-sialylation is responsible for the anti-inflammatory effects of IVIG, thus explaining the high doses of IVIG needed in anti-inflammatory indications. It should be mentioned that all data published by this group to support this claim come from the mouse K/BxN arthritis model [12]–[15]. In addition, supporting data have recently been reported in a mouse model for ITP [21]. In contrast, two groups using different models of ITP failed to show an improved anti-inflammatory effect of SNA+ IgG compared to normal IVIG [18], [22] and no loss of protective function was observed with desialylated IgG in an improved therapeutic model of ITP [22]. Thus, it will be important to investigate the potential contribution of F(ab’)_2_-sialylated IgG molecules within IVIG to anti-inflammation in animal models, particularly the K/BxN or other similar arthritis models. Likewise, further analyses of Fc- and F(ab’)_2_-sialylated IgG in other human *in vitro* systems is warranted.

The anti-inflammatory effect of highly sialylated Fc has been extensively and elegantly demonstrated in several publications [12]–[14]. Most recently, a putative mechanism involving production of IL-33 and secretion of Th2 cytokines by basophils has been convincingly shown [15]. It remains unclear to what extent this mechanism contributes to the overall anti-inflammatory effect of IVIG, which is known to employ a number of different mechanisms to achieve anti-inflammatory effects, including inhibitory signalling through FcγRIIB [23], induction of Tregs [24], attenuation of complement activation [25], [26], recently described new effects on iNKT cells [27] and TH17 cells [28] as well as many more (reviewed in [6], [9], [11], [16], [29]–[31]). Importantly, the anti-inflammatory effect of highly sialylated Fc in models beyond K/BxN needs to be confirmed. A protective effect of plasma-derived Fc fragments in ITP patients was demonstrated many years ago [32] and very recently Ramakrishna and colleagues have proposed a new anti-inflammatory mechanism of isolated Fc by induction of Tregs and secretion of IL-10 [33]. Similarly, it has been shown by de Groot et al. that peptide sequences found in human IgG1 termed “Tregitopes” activate natural Tregs [34]. Thus, these mechanisms need to be seen in context with the newly proposed mechanism via IL-33/basophils. The power of IVIG in anti-inflammation lies in the multiplicity of its actions and interference with multiple pathways in the inflammatory cascade. The contribution of a single mechanism and more specifically the putative contribution of the minor fraction of Fc-sialylated IgG within IVIG is not yet established in the human system.

## Materials and Methods

### Lectin Affinity Chromatography

Normal IVIG (Privigen®, CSL Behring AG, Bern, Switzerland) was fractionated by lectin chromatography, using sialic acid specific *Sambucus nigra* agglutinin (SNA), according to the manufacturer’s description (Vector Laboratories, USA). In a typical run, 1 g of IVIG in Tris-buffered saline at pH 7.5 containing 0.1 M CaCl_2_ (TBS-CaCl_2_) was loaded on 90 mL of agarose-linked SNA. After washing with TBS-CaCl_2_, the fraction bound to the SNA column (+SNA IVIG) was eluted with 0.5 M lactose in TBS (elution fraction 1, E1) followed by elution with 0.5 M lactose in 0.2 M acetic acid (elution fraction 2, E2). The flow through fraction (FT) and the two elution fractions E1 and E2 were collected separately (Fig. 1). Enrichment of plasma derived Fc fragments produced from IVIG (Privigen) was performed in a similar manner. 150 mg was loaded on a 20 mL SNA column and the nonbinding flow through (FT) and one pooled eluate (S+Fc) were collected. The fractions from three pooled runs were concentrated and the buffer changed to PBS by diafiltration using centrifugal filter units with 30 kDa cut off (10 kDa for Fc fragments) (Millipore, USA or Sartorius, Germany). Similar to Fc fragments, F(ab’)_2_ fragments were eluted from the SNA column in one pooled fraction resulting from elution with 0.5 M lactose followed by 0.5 M lactose in 0.2 M acetic acid. All fractions were tested for endotoxin contamination using commercial chromogenic LAL tests (Lonza, Switzerland) or with a kinetic turbidimetric LAL assay. Total sialic acid content in IgG was monitored with lectin blot and quantified by HPLC. Glycoanalysis was performed by LC-MS.

### SDS PAGE and Lectin Blotting

The resulting fractions were separated with SDS PAGE using NuPage 10% BisTris gels under non-reducing or reducing conditions (Invitrogen, USA). The gels were stained with colloidal Coomassie (Gelcode, Thermo Scientific, USA) or blotted on nitrocellulose. The blots were blocked with Carbo-Free blocking solution (Vector Laboratories, USA), probed with biotin-SNA (2 g/L, Vector) and AP-streptavidin (1.5 g/L, Invitrogen) and visualised with chromogenic AP conjugate substrate (BioRad, Switzerland).

### Production of IgG Fragments

F(ab’)_2_ fragments were produced by pepsin digestion of IVIG (Privigen). IVIG was digested with pepsin (0.5 mg/g IgG; Sigma-Aldrich, Switzerland) in acetate buffer pH 4.0 for 2 hours at 37°C. The reaction was stopped by adding 2 M Tris base until a pH of 8 was reached.

Fc fragments were prepared from IVIG (Privigen) by papain digestion and purification by ion exchange and size exclusion chromatography. Remaining Fab was eliminated by running over an Fab-specific affinity chromatography (Athens Research Technologies, USA). Finally, Fc was polished by EndoTrapHD (Hyglos, Germany) resulting in endotoxin levels below 0.05 EU/mg.

Elimination of small digestion products followed by concentration and buffer exchange to PBS was performed by diafiltration using Vivaspin 10 kDa (Fc) or 30 kDa cut off (Fab) spin devices (Sartorius, Germany).

### Enzymatic Desialylation with Neuraminidase

IgG and fragments were desialylated by enzymatic digestion with recombinant neuraminidase from *Clostridium perfringens* expressed in *E. coli* (New England BioLabs, USA) to yield NAase IVIG, NAase E2 and NAase Fc. IgG or Fc was incubated with 7 units enzyme (provider specific) per mg protein for 24 h at 37°C (48 h for E2) followed by concentration and buffer exchange to PBS by diafiltration using centrifugal filter units with 30 kDa cut off (10 kDa for Fc) (Sartorius). The digestion was monitored by sialic acid detection using HPLC or LC-MS.

### Total Sialic Acid Determination by HPLC

Total sialic acid was released by acidic hydrolysis of neuraminic acid in 0.25 M NaHSO_4_ followed by derivatization of the glycan with the fluorophore1,2-diamino-4,5-methylenedioxybenzene dihydrochloride (DMB) (method adapted from [35]). Quantification of the derivatized sialic acid was performed by reverse phase high performance liquid chromatography (RP-HPLC) using N-acetyl neuraminic acid (Neu5Ac; Fluka, Switzerland) as a standard and expressed Neu5Ac per IgG or fragments (F(ab’)_2_ or Fc) (mol/mol or weight/weight). IgG fragments from different lectin fractions have been produced by IdeS digestion using agarose coupled enzyme (FragIT; Genovis, Sweden). Pure F(ab’)_2_ fragments were collected in the flowthrough fraction of the capture select human Fc affinity matrix (BAC, The Netherlands). Monomeric Fc fragments were eluted from the matrix with 0.1 M glycine pH 2.8. To avoid potential contamination by non digested IgG, Fc was further polished using capture select IgG CH1 affinity matrix (BAC). The purity of the fragments was confirmed by SDS-PAGE and Western blotting.

### Glycopeptide and Glycan Analysis by LC-MS

#### Tryptic digestion

A 250 µg portion of IgG was denatured and reduced by addition of 50 µL 6 M guanidine HCl and 2.5 µL 200 mM dithiothreitol (DTT) and incubation at 90°C for 20 minutes. The sample was alkylated by addition of 10 µL 200 mM iodoacetamide and incubation for 30 minutes at room temperature in the dark, followed by addition of 2.5 µL 200 mM DTT and further incubation for 30 minutes in the dark. Samples were digested by addition of 5 µL 1 mg/mL trypsin and 300 µL 100 mM NH_4_HCO_3_ and incubation at 37°C overnight. Following digestion, 2 µL of formic acid were added to each reaction. Tryptic peptides were desalted on homemade C18 spin columns containing 200 mg C18 resin and dried in a vacuum centrifuge.

#### Determination of Fc specific glycoprofile

Dried IgG tryptic peptides were resuspended in 0.1% formic acid and analyzed by liquid chromatography-mass spectrometry (LC-MS) on an Agilent 1200 HPLC coupled to an Agilent 6520 ESI-QTOF. Tryptic peptides from 5 µg of IgG were separated on a reverse phase column (Agilent Poroshell 300SB-C18, 1.0×75 mm, 5 µm) using a gradient from 98% mobile phase A (H_2_O +0.1% formic acid), 2% mobile phase B (acetonitrile +0.1% formic acid) to 12.4% mobile phase B in 12 minutes at a flow rate of 100 µL/min and 40°C. The mobile phase was adjusted to 100% B in 5 minutes, and the column was washed for 3 minutes prior to a 5 minute re-equilibration at the starting conditions. The QTOF was operated in positive polarity with a capillary voltage of 4.0 kV and a fragmentor voltage of 170 V. Chromatograms were extracted and integrated (Fig. S3) for the summed [M+2H]^2+^ and [M+3H]^3+^ ions of each Fc peptide/glycan combination (extraction range of −0.1 to +0.9 m/z, see Table S1) and the areas were used to calculate the relative percent of each glycan present on each peptide.

#### Glycan Release and Conversion to Alditols

Dried tryptic peptides from 250 µg IgG were resuspended in 100 µL 50 mM NH_4_HCO_3_. To deglycosylate, 0.5 µL PNGase F (New England Biolabs, 50,000 U/mL) was added to each sample, and reactions were incubated at 37°C overnight. Peptides were removed from the reactions by passing the sample through a C18 spin column and collecting the glycan containing flow through, which was dried in a vacuum centrifuge. Glycans were converted into alditols after resuspension in 20 µL deionised water by reduction with 200 µL 1 M NaBH_4_, 0.1 M NaOH at 45°C overnight in a vented tube. After addition of 20 µL acetic acid to quench remaining NaBH_4_, the sample was cleaned up by passing through a homemade cation exchange spin tube (200 mg Dowex 50WX4-400, Sigma-Aldrich) and collecting the flow through. Samples were dried in a vacuum centrifuge, followed by repeated resuspension and evaporation of 100 µL portions of 1% acetic acid in methanol to remove boric acid, then reconstituted in mobile phase C (10 mM NH_4_HCO_3_).

#### Glycan Alditol Analysis by LC-MS

Glycan alditols derived from 100 µg of IgG were separated on a graphitized carbon column (Thermo Scientific Hypercarb, 2.1×50 mm, 3 µm) using a gradient from 95% mobile phase C, 5% mobile phase D (10 mM NH_4_HCO_3_ in 80/20 acetontrile/H_2_O) to 60% C, 40% D in 12.5 minutes at 500 µL/min and 40°C. The column was then washed for 2 minutes with 100% D and re-equilibrated at the starting conditions for 2.5 minutes. The QTOF was operated in negative polarity with a capillary voltage of 3.5 kV and a fragmentor setting of 140 V. Chromatograms were extracted and integrated (Figure S3) for each identified glycan alditol (Table S2), summing the signals from the [M-1H]^1^−, [M-2H]^2^−, and [M-3H]^3^− signals (extraction range of −0.1 to +0.9 m/z). The relative amount of each glycan alditol was calculated as an area percent of all the total glycan alditol area. The preparation and analysis of the glycan alditols is similar to previously reported work [36].

### Subclass Distribution, Molecular Size and Endotoxin Determination

Subclass distribution of the IVIG fractions was performed by Luminex using the Bioplex isotyping kit (BioRad) and by nephelometry on a BN ProSpec system (Siemens, Germany) using the recommended subclass assays (N AS IgG1/2 and N latex IgG3/4; Siemens, Germany). Molecular size distribution was performed by analytical size exclusion chromatography (SEC) on a TSKgel G3000SWXL 7.8 mm×30 cm column (Tosoh Bioscience, Germany). Endotoxin quantification was performed using commercial chromogenic endpoint or kinetic LAL tests (Lonza) or with a kinetic turbidimetric LAL assay.

### Antibody Titres and Erythrocyte Binding

#### Antibody titres

Concentrations of antibodies against human parvovirus B19 (B19 virus), cytomegalovirus (human herpes virus 5), Epstein-Barr virus capsid antigen (VCA) (human herpes virus 4), varicella zoster virus (human herpes virus 3) and *Haemophilus influenzae* in IVIG fractions were determined using commercial ELISA Kits (Biotrin, Ireland; DRG Diagnostics, Germany; Institut Virion\Serion, Germany; The Binding Site, UK). Antibodies against, measles virus, rubella virus, tetanus toxoid, Epstein-Barr virus nuclear antigen (NA) (ELISA) and antinuclear antibodies (IF on Hep-2 cells) were measured by a certified medical laboratory (Medics Labor, Switzerland).

#### Erythrocyte binding

IVIG, FT, E1 and E2 were assessed for their binding to human red blood cells (RBC) of healthy volunteer donors of blood groups A (2 donors, #2 & ref), B (2 donors, #12 & #13) and AB (1 donor, #21) (Blutspendedienst SRK Bern, BSD - Blood Transfusion Service SRC Bern, Switzerland). Frozen RBCs were thawed in 0.9% NaCl, containing 880 mM d-sorbitol (Merck, Germany) and washed 3 times (centrifugation for 10 min, 300 g) in Hank̀s buffered salt solution (w/o Ca^++^/Mg^++^; Bioconcept, Switzerland), containing 1 mg/mL BSA (Sigma-Aldrich) (HBSA-1). For the assay, cells were diluted to 1×10^7^ RBCs/mL HBSA-1. Blocking of unspecific binding, was performed by prior addition of human AB serum, diluted 1∶4 in HBSA-1 (BSD), to each tube. The IVIG fractions, all diluted to 8 mg/mL in HBSA-1, were then added to the AB serum and incubated for 30 min at RT. Next, 100 µL of the RBC suspensions (all at 1×10^7^ RBC/mL) were added and the mixtures were incubated for 1 h at RT. After washing 3 times with HBSA-1, the RBC were incubated with a biotinylated anti-Hu IgG mAb (5 µg/mL in HBSA-1; Nordic Immunology, The Netherlands) for 30 min at 4°C. The cells were then washed twice and bound anti-Hu IgG mAb was detected by flow cytometry (FACS analysis) using phycoerythrin-labelled streptavidin (5 µg/mL; Jackson Immuno Research, UK) on a BD FACS Canto II cell sorter. 10̀000 cells were acquired and the data was analysed using BD FACS Diva Software (Becton Dickinson, Switzerland). Δ MFI was calculated by subtracting the MFI of RBC stained without addition of IVIG fractions.

### Whole Blood Stimulation Assay

Blood was donated voluntarily with signed informed consent under medical supervision. The donation procedure has been approved by an in-house ethical committee led by the medical direction. Heparinised whole blood from healthy volunteer donors was stimulated with 10 mg/L phytohemagglutinin-M (PHA, Roche diagnostics, Switzerland) or 100 ng/L LPS (*E. Coli* O111:B4; Sigma-Aldrich). Different IVIG fractions were added to the whole blood simultaneously with stimulating agent, leading to 50% concentrated whole blood in PBS (no differences observed to dilutions made with cell culture medium). In control samples, whole blood was incubated with addition of IgG but without stimulation. After 20±2 h of incubation at 37°C and 5% CO_2_, cells and supernatants were harvested for further analysis. The cells were centrifuged and then resuspended in PBS. Then cells were incubated with PE-labelled anti-CD54, FITC-labelled anti-CD14 and PerCP-labelled anti-CD45 (BD Biosciences, USA) for 30 min at 4°C. After red blood cell lysis with BD Pharm Lyse (BD Biosciences) the cells were taken up in PBS pH 7.4. CD54 expression levels on monocytes were determined by flow cytometry on a FACS Canto II (BD Biosciences). MCP-1 was quantified in cell culture supernatants by ELISA (DuoSet, R&D Systems, USA).

## Supporting Information

Figure S1 **Total sialic acid quantification in Fc and Fab regions of IVIG fractions.** A: Total sialic acid content in single chain Fc fragments produced from the indicated IVIG lectin fractions by IdeS digestion and quantified by HPLC. B: Total sialic acid content in F(ab’)_2_ fragments produced from the indicated IVIG fractions. C and D: Control of the purity of the IdeS fragments by SDS PAGE and coomassie staining. lane 1: IVIG, lane 2: FT, lane 3: E1, lane 4: E2, lane 5: NAase IVIG, M: molecular weight marker.
(TIFF)

Figure S2 **IgG Subclass distribution in the different IVIG fractions.** IgG subclasses have been determined with Luminex and Nephelometry. Results show the relative subclass content in the indicated IVIG fractions (mean values of both methods).
(TIFF)

Figure S3 **Extracted ion chromatograms for Fc-glycoptides and glycan alditols.** *Top panel:* Example of extracted ion chromatograms for six of the seven IgG1 Fc-derived glycopeptides. A description of the glycan representations and calculated masses of the glycopeptides can be found in Table S1. *Bottom panel:* Example of extracted ion chromatograms for the identified glycan alditols released from a sample of IgG. The glycan alditols and their masses are described in Table S2. Glycan representations are drawn according to the legend in Table S1. The peak labelled with an asterisk (*) was not glycan related. Glycan representations are described in Table S1.
(TIFF)

Table S1 **Peptide sequence, proposed glycan structure, and calculated monoisotopic masses of identified IgG Fc tryptic glycopeptides.**
(DOC)

Table S2 **Proposed structure, molecular formula, calculated monoisotopic mass, and calculated m/z values of the alditol forms of identified glycans released from IgG.**
(DOC)
