# Supplementary figures and images for: Analysis and Functional Consequences of Increased Fab-Sialylation of Intravenous Immunoglobulin (IVIG) after Lectin Fractionation
Source: PLoS One. 2012 Jun 4;7(6):e37243. doi: 10.1371/journal.pone.0037243 (PMC3366990; doi:10.1371/journal.pone.0037243)

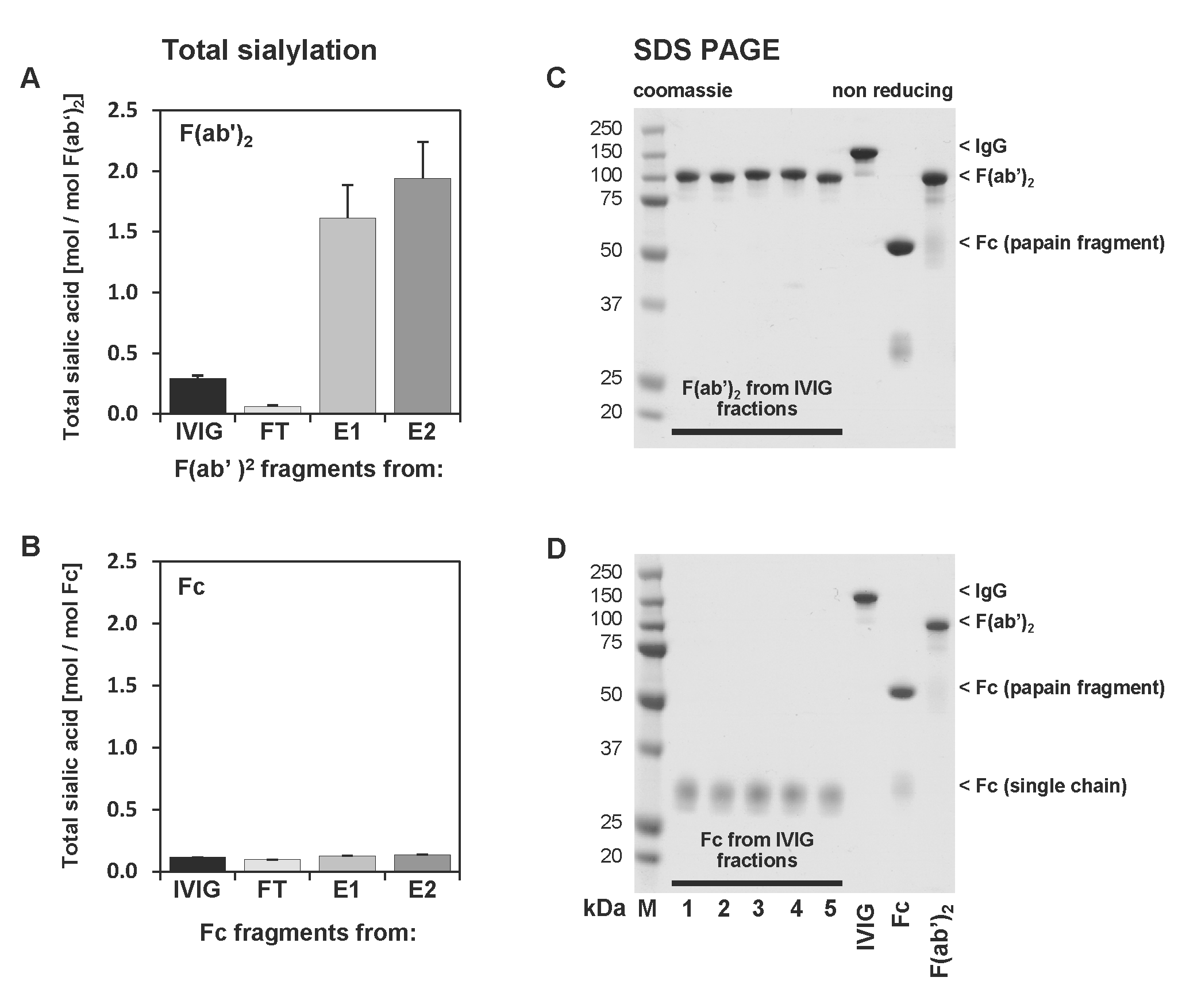

Supplement: Figure S1 — Total sialic acid quantification in Fc and Fab regions of IVIG fractions. A: Total sialic acid content in single chain Fc fragments produced from the indicated IVIG lectin fractions by IdeS digestion and quantified by HPLC. B: Total sialic acid content in F(ab’)2 fragments produced from the indicated IVIG fractions. C and D: Control of the purity of the IdeS fragments by SDS PAGE and coomassie staining. lane 1: IVIG, lane 2: FT, lane 3: E1, lane 4: E2, lane 5: NAase IVIG, M: molecular weight marker. (TIFF) [file pone.0037243.s001.tif]

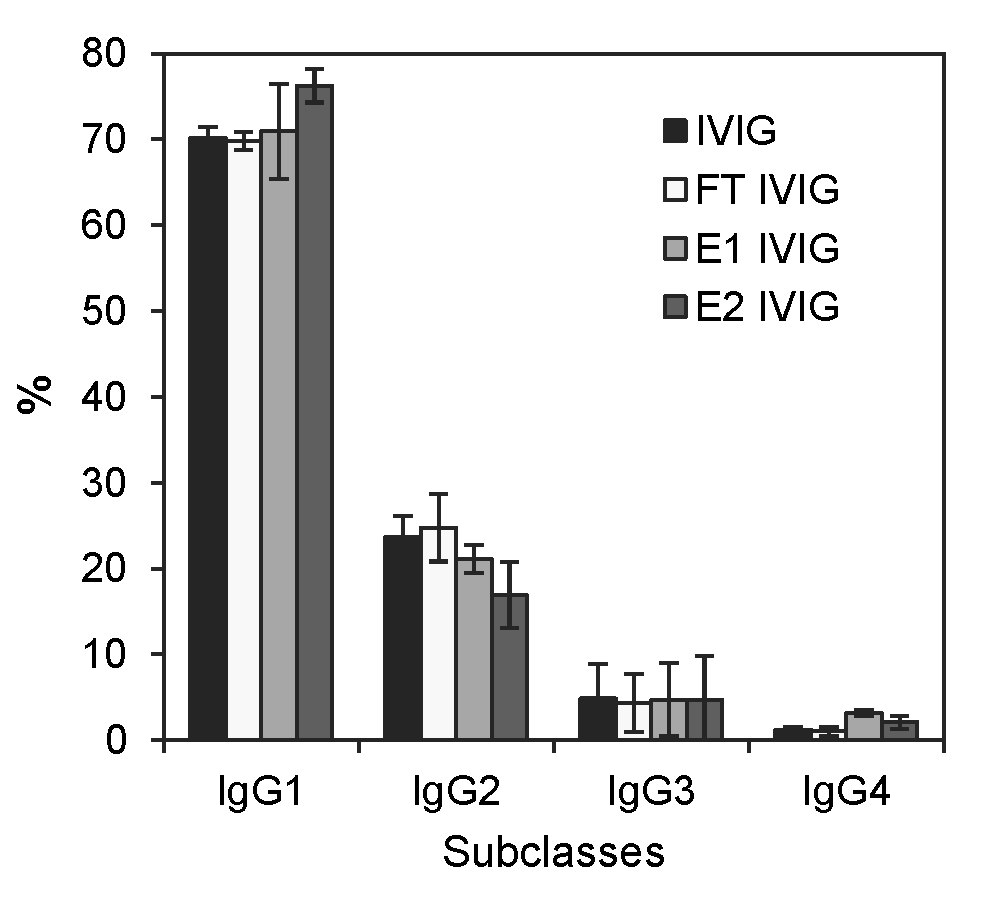

Supplement: Figure S2 — IgG Subclass distribution in the different IVIG fractions. IgG subclasses have been determined with Luminex and Nephelometry. Results show the relative subclass content in the indicated IVIG fractions (mean values of both methods). (TIFF) [file pone.0037243.s002.tif]

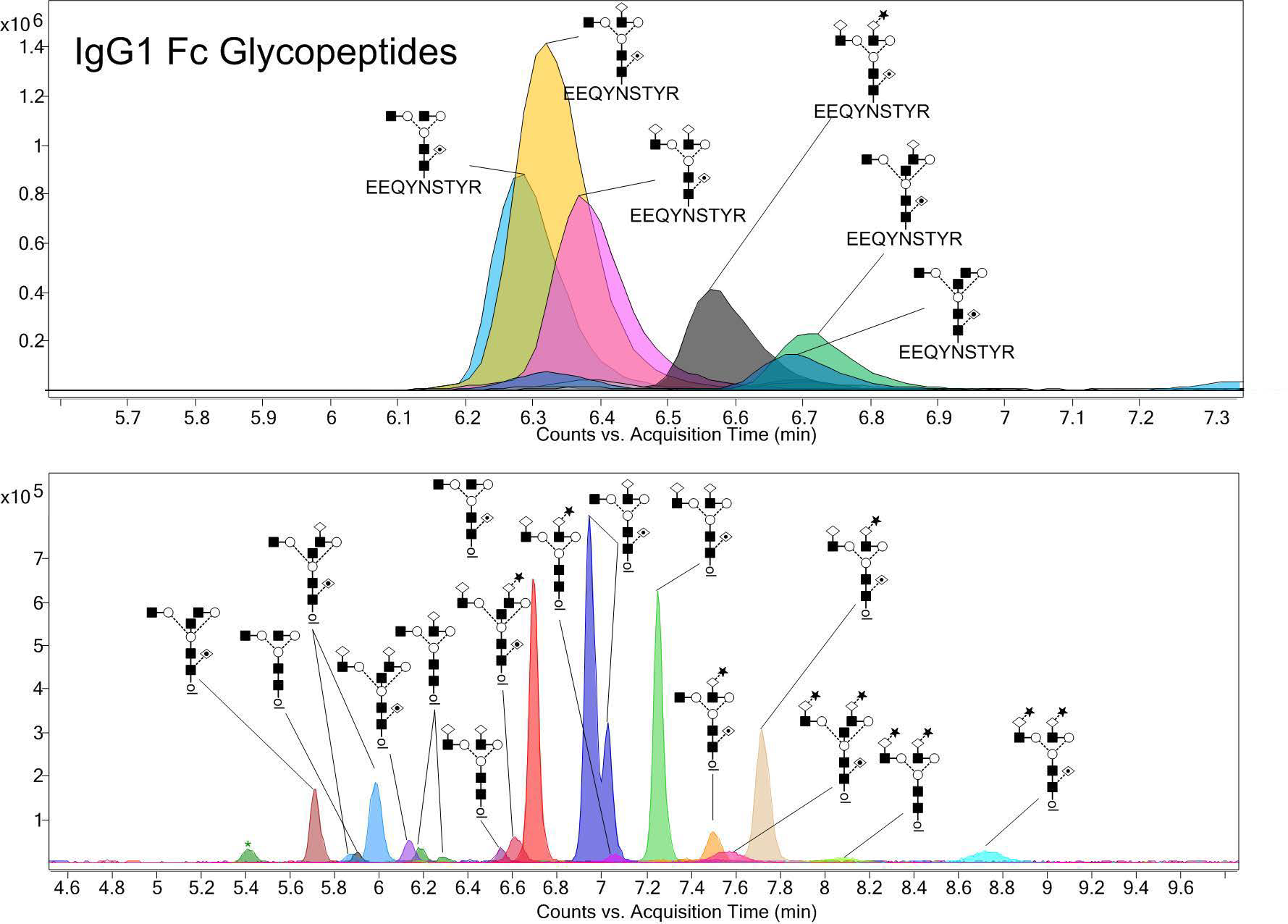

Supplement: Figure S3 — Extracted ion chromatograms for Fc-glycoptides and glycan alditols. Top panel: Example of extracted ion chromatograms for six of the seven IgG1 Fc-derived glycopeptides. A description of the glycan representations and calculated masses of the glycopeptides can be found in Table S1. Bottom panel: Example of extracted ion chromatograms for the identified glycan alditols released from a sample of IgG. The glycan alditols and their masses are described in Table S2. Glycan representations are drawn according to the legend in Table S1. The peak labelled with an asterisk (*) was not glycan related. Glycan representations are described in Table S1. (TIFF) [file pone.0037243.s003.tif]
