# Supplementary material for: Analysis and Functional Consequences of Increased Fab-Sialylation of Intravenous Immunoglobulin (IVIG) after Lectin Fractionation
Source: PLoS One. 2012 Jun 4;7(6):e37243. doi: 10.1371/journal.pone.0037243 (PMC3366990; doi:10.1371/journal.pone.0037243)
Supplement: Table S1 — Peptide sequence, proposed glycan structure, and calculated monoisotopic masses of identified IgG Fc tryptic glycopeptides. (DOC) [file pone.0037243.s004.doc]

**Table S1.** Peptide sequence, proposed glycan structure, and calculated monoisotopic masses of identified IgG Fc tryptic glycopeptides.

| **IgG Subclass** | **Peptide Sequence** | **Proposed Glycan Structure** | **Glycan** | **Mass (Da)** | **[M+2H]^2+^ (m/z)** | **[M+3H]^3+^ (m/z)** |
| --- | --- | --- | --- | --- | --- | --- |
|  |  |  | FA2G0 | 2633.0386 | 1317.5266 | 878.6868 |
|  |  |  | FA2G1 | 2795.0914 | 1398.5530 | 932.7044 |
|  |  |  | FA2G2 | 2957.1442 | 1479.5794 | 986.7220 |
| IgG1 | EEQYNSTYR |  | FA2G2S1 | 3248.2396 | 1625.1271 | 1083.7538 |
|  |  |  | FA2G2S2 | 3539.3350 | 1770.6748 | 1180.7856 |
|  |  |  | FA2BG0 | 2836.1180 | 1419.0663 | 946.3800 |
|  |  |  | FA2BG1 | 2998.1708 | 1500.0927 | 1000.3976 |
|  |  |  | FA2G0 | 2601.0488 | 1301.5317 | 868.0235 |
|  |  |  | FA2G1 | 2763.1016 | 1382.5581 | 922.0411 |
|  |  |  | FA2G2 | 2925.1544 | 1463.5845 | 976.0587 |
| IgG2/IgG3 | EEQFNSTFR |  | FA2G2S1 | 3216.2498 | 1609.1322 | 1073.0905 |
|  |  |  | FA2G2S2 | 3507.3452 | 1754.6799 | 1170.1223 |
|  |  |  | FA2BG0 | 2804.1282 | 1403.0714 | 935.7167 |
|  |  |  | FA2BG1 | 2966.1810 | 1484.0978 | 989.7343 |

IgG2 and IgG3 share a common peptide sequence [37], and the resulting tryptic glycopeptides cannot be differentiated. Glycan structures are abbreviated according the system proposed by the Oxford glycobiology institute [38] as follows: all proposed glycan structures contain a trimanosyl core; presence of a core fucose is indicated by F; A2 indicates a biantennary glycan with two GlcNAc antenna; Gx indicates the number (x) of glucose on the antenna; Sx indicates the number (x) of sialic acids on the antenna; B indicates a bisecting GlcNAc. Glycan structures are represented according to the conventions proposed by Harvey, et al. [39]. In the case where the observed mass corresponds to multiple possible isomeric structures, only a single structure is represented for simplicity.

37. Wuhrer M, Stam JC, van de Geijn FE, Koeleman CA, Verrips CT, et al. (2007) Glycosylation profiling of immunoglobulin G (IgG) subclasses from human serum. Proteomics 7: 4070-4081.

38. Guile GR, Rudd PM, Wing DR, Prime SB, Dwek RA (1996) A Rapid High-Resolution High-Performance Liquid Chromatographic Method for Separating Glycan Mixtures and Analyzing Oligosaccharide Profiles. Analytical Biochemistry 240: 210-226.

39. Harvey DJ, Merry AH, Royle L, Campbell MP, Dwek RA, et al. (2009) Proposal for a standard system for drawing structural diagrams of N- and O-linked carbohydrates and related compounds. Proteomics 9: 3796-3801.
