# Supplementary material for: Analysis and Functional Consequences of Increased Fab-Sialylation of Intravenous Immunoglobulin (IVIG) after Lectin Fractionation
Source: PLoS One. 2012 Jun 4;7(6):e37243. doi: 10.1371/journal.pone.0037243 (PMC3366990; doi:10.1371/journal.pone.0037243)
Supplement: Table S2 — Proposed structure, molecular formula, calculated monoisotopic mass, and calculated m/z values of the alditol forms of identified glycans released from IgG. (DOC) [file pone.0037243.s005.doc]

**Table S2.** Proposed structure, molecular formula, calculated monoisotopic mass, and calculated m/z values of the alditol forms of identified glycans released from IgG.

| **Glycan** | **Proposed Glycan Alditol Structure** | **Alditol Molecular Formula** | **Alditol Mass (mono., Da)** | **[M-1H]^1-^ (m/z)** | **[M-2H]^2-^ (m/z)** | **[M-3H]^3-^ (m/z)** |
| --- | --- | --- | --- | --- | --- | --- |
| A2G0 |  | C_50_H_86_N_4_O_36_ | 1318.5022 | 1317.4949 | 658.2438 | 438.4935 |
| FA2G0 |  | C_56_H_96_N_4_O_40_ | 1464.5601 | 1463.5528 | 731.2728 | 487.1794 |
| A2G1 |  | C_56_H_96_N_4_O_41_ | 1480.5550 | 1479.5477 | 739.2702 | 492.5111 |
| FA2G1 |  | C_62_H_106_N_4_O_45_ | 1626.6129 | 1625.6056 | 812.2992 | 541.1970 |
| A2G2 |  | C_62_H_106_N_4_O_46_ | 1642.6078 | 1641.6005 | 820.2966 | 546.5287 |
| FA2BG0 |  | C_64_H_109_N_5_O_45_ | 1667.6395 | 1666.6322 | 832.8125 | 554.8726 |
| A2BG1 |  | C_64_H_109_N_5_O_46_ | 1683.6344 | 1682.6271 | 840.8099 | 560.2042 |
| FA2G2 |  | C_68_H_116_N_4_O_50_ | 1788.6657 | 1787.6584 | 893.3256 | 595.2146 |
| FA2BG1 |  | C_70_H_119_N_5_O_50_ | 1829.6923 | 1828.6850 | 913.8389 | 608.8902 |
| A2BG2 |  | C_70_H_119_N_5_O_51_ | 1845.6872 | 1844.6799 | 921.8363 | 614.2218 |
| FA2G1S1 |  | C_73_H_123_N_5_O_53_ | 1917.7083 | 1916.7010 | 957.8469 | 638.2288 |
| A2G2S1 |  | C_73_H_123_N_5_O_54_ | 1933.7032 | 1932.6959 | 965.8443 | 643.5605 |
| FA2BG2 |  | C_76_H_129_N_5_O_55_ | 1991.7451 | 1990.7378 | 994.8653 | 662.9078 |
| FA2G2S1 |  | C_79_H_133_N_5_O_58_ | 2079.7611 | 2078.7538 | 1038.8733 | 692.2464 |
| A2G2S2 |  | C_84_H_140_N_6_O_62_ | 2224.7986 | 2223.7913 | 1111.3920 | 740.5923 |
| FA2BG2S1 |  | C_87_H_146_N_6_O_63_ | 2282.8405 | 2281.8332 | 1140.4130 | 759.9396 |
| FA2G2S2 |  | C_90_H_150_N_6_O_66_ | 2370.8565 | 2369.8492 | 1184.4210 | 789.2782 |
| FA2BG2S2 |  | C_98_H_163_N_7_O_71_ | 2573.9359 | 2572.9286 | 1285.9607 | 856.9714 |

Glycan abbreviations and representations are described in Table S1.
